# Effect Of treatment on serum C3, C4, IL-10, IL1-β, IL-6, TNF-α in patients with systemic lupus erythematosus

**DOI:** 10.5937/jomb0-62357

**Published:** 2026-06-16

**Authors:** Nadira Reyimu, Yueyue Jing

**Affiliations:** 1 The Second Affiliated Hospital of Xinjiang Medical University, Department of of Cardiovascular, Urumqi, Xinjiang, China; 2 Yan'an People's Hospital, Department of Cardiovascular Medicine, Yan'an, Shaanxi, China

**Keywords:** serum complement C3, complement C4, IL-10, IL1-β, IL-6, TNF-α, systemic lupus erythematosus, Libman-Sacks endocarditis, lifestyle optimisation, nutritional interventions, exercise training, serumski komplement C3, komplement C4, IL-10, IL1-β, IL-6, TNF-α, sistemski eritemski lupus, Libman-Sacks endokarditis, optimizacija životnog stila, nutritivne intervencije, fizički trening

## Abstract

**Background:**

This study aimed to evaluate the effects of structured lifestyle optimisation, including nutritional intake and exercise training, on serum levels of Complement C3, C4, IL-10, IL1-<span style="color: rgb(32, 33, 34); font-family: sans-serif; font-size: 16px; background-color: rgb(248, 249, 250);">β</span>, IL-6, and TNF-<span style="color: rgb(32, 33, 34); font-family: sans-serif; font-size: 16px; background-color: rgb(248, 249, 250);">α</span> in patients with systemic lupus erythematosus (SLE) and Libman-Sacks endocarditis (LSE).

**Methods:**

A total of 122 SLE patients with LSE, treated at our hospital from May 2022 to May 2024, were randomised into a control group (CG, n = 61) receiving conventional medical interventions and an intervention group (IG, n = 61) receiving additional personalised exercise and nutritional interventions for 24 weeks. Serum levels of Complement C3, C4, IL-10, IL1-<span style="color: rgb(32, 33, 34); font-family: sans-serif; font-size: 16px; background-color: rgb(248, 249, 250);">β</span>, IL-6, and TNF-<span style="color: rgb(32, 33, 34); font-family: sans-serif; font-size: 16px; background-color: rgb(248, 249, 250);">α</span> were measured at baseline, 3 months, and 6 months. Secondary endpoints included nutritional status (as measured by albumin and haemoglobin levels) and patient compliance.

**Results:**

Of 113 patients completing the study, the intervention group (IG) showed significant improvement in serum Complement C3 (0.84 ± 0.18 g/L vs. 0.75± 0.16 g/L in the control group [CG], P= 0.006) and albumin levels (37.12± 5.37 g/L vs. 35.00 ± 4.89 g/L, P= 0.03) at 6 months. Complement C4 levels increased in the IG (0.20 ± 0.09 g/L vs. 0.17± 0.12 g/L, P= 0.136, not significant). IL-1<span style="color: rgb(32, 33, 34); font-family: sans-serif; font-size: 16px; background-color: rgb(248, 249, 250);">β</span> (3 .9 ± 3 .7 7 pg/mL vs. 4.6± 4.1 pg/mL, P= 0.468) and TNF-<span style="color: rgb(32, 33, 34); font-family: sans-serif; font-size: 16px; background-color: rgb(248, 249, 250);">α</span> (12.6± 4.1 pg/mL vs. 14.1 ±5.2 pg/mL, P= 0.092) showed non-significant reductions in the IG compared to the CG. No significant changes were observed in IL-10 or IL-6 levels. Compliance was significantly higher in the IG (92.86% vs. 78.95% , P= 0.038).

**Conclusions:**

Structured lifestyle optimisation significantlyimproved serum Complement C3 and albumin levels andshowed non-significant trends toward reductions in IL-1<span style="color: rgb(32, 33, 34); font-family: sans-serif; font-size: 16px; background-color: rgb(248, 249, 250);">β</span> and TNF-<span style="color: rgb(32, 33, 34); font-family: sans-serif; font-size: 16px; background-color: rgb(248, 249, 250);">α</span> in SLE patients with LSE, suggesting a potentialadjunctive role in managing immune and nutritional status.

## Abstract (sr)

**Uvod:**

Cilj ove studije je bio da proceni efekte struktuirane optimizacije životnog stila, uključujući unos hrane i fizičke vežbe, na serumske nivoe komplementa C3, C4, IL-10, IL1-<span style="color: rgb(32, 33, 34); font-family: sans-serif; font-size: 16px; background-color: rgb(248, 249, 250);">β</span>, IL-6 i TNF-<span style="color: rgb(32, 33, 34); font-family: sans-serif; font-size: 16px; background-color: rgb(248, 249, 250);">α</span> kod pacijenata sa sistemskim eritemskim lupusom (SLE) i Libman-Sacks endokarditisom (LSE).

**Metode:**

Ukupno 122 pacijenta sa SLE i LSE, lečena u našoj bolnici u periodu od maja 2022. do maja 2024. godine, su bila nasumično raspoređena u kontrolnu grupu (KG, n = 61), koja je dobijala uobičajene medicinske intervencije, i intervencionu grupu (IG, n = 61), koja je tokom 24 nedelje dobijala dodatne personalizovane intervencije u vidu fizičkih vežbi i nutritivne podrške. Serumski nivoi komplementa C3, C4, IL-10, IL1-<span style="color: rgb(32, 33, 34); font-family: sans-serif; font-size: 16px; background-color: rgb(248, 249, 250);">β</span>, IL-6 i TNF-<span style="color: rgb(32, 33, 34); font-family: sans-serif; font-size: 16px; background-color: rgb(248, 249, 250);">α</span> mereni su na početku, nakon 3 meseca i nakon 6 meseci. Sekundarne krajnje tačke su obuhvatale nutritivni status (meren preko nivoa albumina i hemoglobina) i stepen pridržavanja pacijenata.

**Rezultati:**

Od 113 pacijenata koji su završili studiju, grupa gde je sa intervencijom (IG) je pokazala značajno poboljšanje nivoa serumskog komplementa C3 (0,84 ± 0,18 g/L naspram 0 ,7 5 ± 0 ,1 6 g/L u kontrolnoj grupi [KG ], P= 0,006) i nivoa albumina (37,12± 5,37 g/L naspram 35,00 ± 4,89 g/L, P= 0,03) nakon 6 meseci. Nivoi komplementa C4 povećali su se u IG (0,20 ± 0,09 g/L naspram 0,17± 0,12 g/L, P= 0,136, nije značajno). IL-1<span style="color: rgb(32, 33, 34); font-family: sans-serif; font-size: 16px; background-color: rgb(248, 249, 250);">β</span> (3,9± 3,77 pg/mL naspram 4,6± 4,1 pg/mL, P= 0,468) i TNF-<span style="color: rgb(32, 33, 34); font-family: sans-serif; font-size: 16px; background-color: rgb(248, 249, 250);">α</span> (12,6± 4,1 pg/mL naspram 14,1± 5,2 pg/mL, P= 0,092) pokazali su neznačajno smanjenje u IG u poređenju sa KG. Nisu zabeležene značajne promene u nivoima IL-10 ili IL-6. Pridržavanje pacijenata je bilo značajno veće u IG (92,86% naspram 78,95% , P= 0,038).

**Zaključak:**

Struktuirana optimizacija životnog stila jeznačajno poboljčala nivoe serumskog komplementa C3 ialbumina i pokazala neznačnajne trendove ka smanjenjuIL-1<span style="color: rgb(32, 33, 34); font-family: sans-serif; font-size: 16px; background-color: rgb(248, 249, 250);">β</span> i TNF-<span style="color: rgb(32, 33, 34); font-family: sans-serif; font-size: 16px; background-color: rgb(248, 249, 250);">α</span> kod pacijenata sa SLE i LSE, sto ukazuje napotencijalnu pomocnu ulogu u upravljanju imunoloskim inutritivnim statusom.

## Introduction

Systemic lupus erythematosus (SLE) is a diffuse connective tissue disease with multi-organ and multi-system involvement. The prevalence of SLE in China is 30-70 per 100,000 population, and SLE is highly prevalent in women of reproductive age [1]. Evidence suggests that the pathogenesis of SLE may be related to immunological abnormalities, endocrinological factors, genetics, infections, and other factors [2]. SLE is often associated with cardiovascular disease, as well as involvement in the kidneys, skin, joints, and other organs. Epidemiological studies have shown that more than 50% of SLE patients in China are associated with cardiac damage [3], and myocardium, pericardium, valves, coronary atherosclerosis, and conduction system can be involved. The early stage of cardiac damage is insidious, and clinical attention is not paid to it, and it develops to the late stage of the disease with adverse consequences. Cardiovascular disease has become the third leading cause of death in patients with SLE after infection and renal failure [4]. Liebman-Sacks endocarditis (LSE), also known as non-bacterial thrombotic endocarditis, is a typical cardiac manifestation of SLE, characterised by the formation of non-infectious yet mounting, cumbersome organisms of the valves [5]. It has been found that LSE is associated with 1 in 10 patients with SLE; thus, its incidence is not uncommon and has been reported in up to 74% of autopsy reports [6][7]. If left untreated, it can lead to the formation of heart failure, the end stage of cardiovascular disease development, which is a serious threat to human health and life safety.

Several studies have shown that lifestyle interventions can enhance the quality of life for SLE patients [8]. As SLE is a chronic autoimmune disease, patients have a low immune system and present with fatigue, tiredness, muscle and joint swelling and pain [9]. Sedentary lifestyle and physical inactivity are risk factors for cardiovascular disease. Exercise training, however, enhances the immune system, thereby improving symptoms and quality of life for patients. O'Dwyer et al.'s study also revealed that exercise interventions have no adverse effects and improve cardiorespiratory fitness while reducing fatigue in patients [10]. In addition, SLE patients are at high risk of malnutrition, especially when organ damage involves the digestive system, which is often accompanied by a series of gastrointestinal symptoms such as abdominal pain, nausea, and intestinal absorption dysfunction, coupled with the energy consumption of connective tissue disease itself, resulting in SLE patients being more likely to suffer from malnutrition. A growing number of national and international studies have shown that SLE patients have different degrees of nutritional problems, including obesity, anaemia, sarcopenia and hypoproteinemia [11][12]. One study counted the effects of nutritional supplementation on SLE over the past 15 years and found that appropriate dietary supplementation improved patients' fatigue, inflammation levels, and disease activity and could be used as an adjunctive treatment for SLE [13].

There are few studies on interventions for LSE patients, so this study applied structured optimisation of nutritional intake and exercise training to LSE patients to investigate whether the intervention improved outcomes and physiological markers in these patients.

## Materials and methods

### Study design

This randomised, controlled, parallel-group clinical trial was conducted at the First Affiliated Hospital of Jinzhou Medical University (Jinzhou, Liaoning, China) and Yan'an People's Hospital (Yan'an, Shaanxi, China) between May 2022 and May 2024. The aim was to assess the effect of a structured lifestyle optimisation program on serum immunological and inflammatory markers in patients with systemic lupus erythematosus (SLE) and echocardiography-confirmed Libman-Sacks endocarditis (LSE). The study was conducted in accordance with the Declaration of Helsinki, and the protocol received approval from the ethics committees of both participating hospitals. Written informed consent was obtained from all participants before their enrollment.

### Participants

A total of 122 patients, aged between 18 and 60 years, were included in the study. All participants fulfilled the Systemic Lupus International Collaborating Clinics (SLICC) classification criteria for SLE, confirmed by both clinical evaluation and laboratory testing, and had a diagnosis of LSE verified by transthoracic echocardiography, which showed valvular thickening or vegetations consistent with non-infectious endocarditis. Patients were excluded if they had malignant tumours, psychiatric or cognitive disorders that could affect compliance, severe multi-organ failure, pregnancy or breastfeeding, or recent participation in another clinical trial. Baseline demographic and clinical data, including disease duration, medication use, and Systemic Lupus Erythematosus Disease Activity Index (SLEDAI) scores, were recorded. Randomisation was performed using a computer-generated sequence to allocate participants equally to the control group (CG) and the intervention group (IG).

### Intervention protocol

Patients in the control group received standard medical care according to current rheumatology practice, which included the use of anti-rheumatic agents such as hydroxychloroquine, methotrexate, or azathioprine, as well as corticosteroids, immunosuppressants, and cardiovascular risk-reducing medications, all as clinically indicated. General lifestyle advice was provided during routine outpatient visits in line with hospital-standard patient education procedures.

The intervention group received the same standard medical treatment in addition to a structured lifestyle optimisation program designed by a multidisciplinary team of rheumatologists, cardiologists, physiotherapists, and nutritionists. This program combined an exercise regimen and individualised dietary counselling. Exercise prescriptions were developed after assessing functional capacity, cardiovascular status, and musculoskeletal limitations. Aerobic activities such as brisk walking, cycling, yoga, or tai chi were recommended according to patient preference and tolerance. Sessions were scheduled at least three times per week, each lasting between 30 and 50 minutes, with a target intensity of 50-60% of the age-predicted maximum heart rate (calculated as 220 minus age). Initial training sessions were supervised and monitored using heart rate devices, while later sessions were performed at home with patient-maintained activity logs. The exercise load was progressively increased based on individual tolerance and the absence of adverse effects.

The dietary component of the program consisted of personalised nutritional guidance based on the patient's clinical condition and laboratory findings. Recommended protein intake ranged from 1.0 to 1.2 g per kilogram of body weight per day. Sodium intake was restricted to less than 2 g per day, and dietary fibre intake was encouraged at 25-30 g per day. Vitamin D supplementation (800-1000 IU/day) was provided to all patients due to the high prevalence of deficiency in SLE, along with calcium, magnesium, and other micronutrients when indicated. Photo-sensitising foods, such as citrus fruits and celery, were avoided, while anti-inflammatory foods, including omega-3-rich fish, berries, and green tea, were encouraged. Counselling sessions were conducted face-to-face at baseline, every two months, and at study completion, with interim follow-up via telephone or online consultation.

### Blood sample collection and processing

Fasting venous blood samples (5 mL) were collected at baseline, three months, and six months between 07:00 and 09:00 in the morning. Samples were collected in serum-separating tubes (Becton Dickinson, USA), allowed to clot for 30 minutes at room temperature, and centrifuged at 3000 rpm for 10 minutes at 4°C. The resulting serum was aliquot- ed into polypropylene tubes and stored at -80°C until analysis. All samples from the same participant were analysed in a single batch to reduce inter-assay variability.

### Laboratory measurements

Serum Complement C3 and C4 concentrations were measured by immunoturbidimetry using a Beckman Coulter AU5800 Chemistry Analyser (Beckman Coulter Inc., Brea, CA, USA) with original manufacturer reagents. Daily calibration and two-level internal quality control were performed before testing.

Fasting glucose, total cholesterol (TC), triglycerides (TG), high-density lipoprotein cholesterol (HDL-C), and low-density lipoprotein cholesterol (LDL-C) were measured enzymatically using the same Beckman Coulter AU5800 Chemistry Analyser with dedicated reagents from the manufacturer. Very low-density lipoprotein cholesterol (VLDL-C) was calculated using the Friedewald formula (VLDL-C=TG/2.2, with TG expressed in mmol/L) when TG<4.5 mmol/L.

Interleukin-10 (IL-10), interleukin-1β (IL-1β), interleukin-6 (IL-6), and tumour necrosis factor-α (TNF-α) were quantified using high-sensitivity ELISA kits (R&D Systems, Minneapolis, MN, USA) according to the manufacturer's instructions. Optical density readings were obtained at 450 nm with wavelength correction at 570 nm using a BioTek Synergy HTX multi-mode microplate reader (Agilent Technologies, USA). All assays were performed in duplicate, and mean values were used for analysis.

### Quality control and reference intervals

Internal quality control was ensured through the use of low, medium, and high control sera (Bio-Rad Laboratories, Hercules, CA, USA) at the beginning and end of each analytical run. External quality assessment was conducted through participation in the National Centre for Clinical Laboratories (NCCL) proficiency testing program. Runs in which control values exceeded ±2 standard deviations from the target mean were repeated. Reference intervals, based on manufacturer specifications and verified in our laboratory, were as follows: Complement C3, 0.9-1.8 g/L; Complement C4, 0.1-0.4 g/L; IL-10, <5 pg/mL; IL-1β, <5 pg/mL; IL-6, <7 pg/mL; TNF-α, <15 pg/mL.

### Outcomes

The primary outcome was the change in serum Complement C3, C4, IL-10, IL-1β, IL-6, and TNF-α from baseline to six months. Secondary outcomes included changes in serum albumin, haemoglobin, patient compliance with the lifestyle program, and selected cardiopulmonary performance parameters, including first- and second-minute heart rate recovery (HRR1 and HRR2) and six-minute walk test (6MWT) distance. Compliance was defined as adherence to at least 80% of the prescribed exercise and dietary recommendations.

### Statistical analysis

Data analysis was performed using SPSS version 21.0 (IBM Corp., Armonk, NY, USA). Normality of continuous variables was assessed using the Shapiro-Wilk test. Normally distributed variables were expressed as mean ± standard deviation and compared between groups using the independent-samples t-test. Non-normally distributed variables, including IL-1β, IL-6, TNF-α, and triglycerides, were expressed as median (interquartile range) and compared using the Mann-Whitney U test. Within-group changes over time were analysed using paired t-tests for normally distributed data or Wilcoxon signed-rank tests for non-normally distributed data. Categorical variables were summarised as frequencies and percentages and compared using the chi-square test or Fisher's exact test. All statistical tests were two-tailed, with p-values of less than 0.05 considered statistically significant.

## Results

### Study population and baseline characteristics

**Figure 1:**
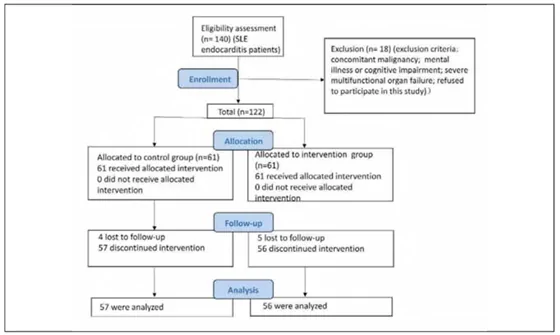
Comparison of Treg, TGF-β1 between AR and AR+AH groups (Error bars: SD; n = 127 for AR, n = 134 for AR+AH). (A) Comparison of Tregs. (B) Comparison of TGF-β1. (C) Diagnosing AH complications in AR pediatric patients through Treg and TGF-β detection.

**Figure 2:**
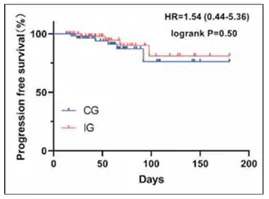
Progression-free survival at six months in both groups.

**Figure 3:**
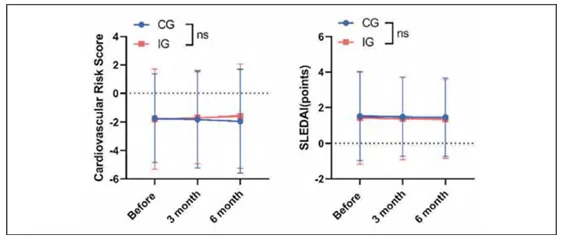
Comparison of cardiovascular risk scores and disease activity within six months of intervention between the two groups.

**Figure 4:**
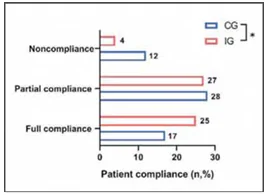
Comparison of adherence between the two groups, *P<0.05.

**Figure 5:**
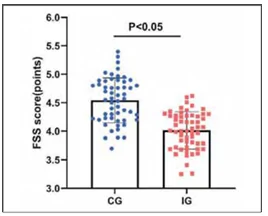
Scores of fatigue severity six months after the intervention in both groups of patients.

**Figure 6:**
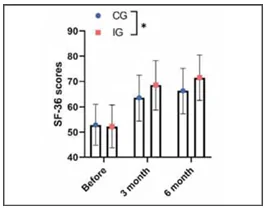
Quality of life scores in both groups six months after the intervention, *P<0.05.

As shown in the trial profile (Figure 1), 122 patients with systemic lupus erythematosus (SLE) and echocardiography-confirmed Libman-Sacks endocarditis (LSE) were enrolled in this randomised controlled trial conducted from May 2022 to May 2024. Of these, 113 patients completed the study, with 4 withdrawals in the control group (CG, n = 57) and 5 in the intervention group (IG, n = 56) due to personal reasons, such as relocation or inability to attend follow-up sessions (Figure 1). The mean age of completers was 37.92±10.76 years (range: 22-45 years). Baseline characteristics, including age, sex, SLE duration, Systemic Lupus Erythematosus Disease Activity Index (SLEDAI) scores, body mass index (BMI), lipid profiles, and medication use (e.g., hydroxychloroquine, corticosteroids, immunosuppressants), were comparable between the CG and IG, with no statistically significant differences (P>0.05 for all comparisons, Table 1). This ensured that observed differences in outcomes were attributable to the structured lifestyle intervention rather than baseline imbalances. Figure 2 Figure 3 Figure 4 Figure 5 Figure 6

**Table 1:** Baseline characteristics of patients (mean ± standard deviation) or n (%). BMI, body mass index; DBP diastolic blood pressure; SBFJ systolic blood pressure; HDL, high-density lipoproteins; TG, triglyceride; DMARDs, disease-modifying anti-rheumatic drugs; NSAIDs, nonsteroidal anti-inflammatory drugs.

Parameter	Control	PCT	X2(t)	P
Number	57	56	-	-
Age (years)	39.2±9.15	37.6±8.89	0.943	0.348
Gender, n (%) Female	53 (92.98)	54 (96.43)	0.667	0.414
Male	4 (7.02)	2 (3.57)		
BMI (kg/m^2^)	28.87±3.64	29.04±3.92	0.239	0.812
Marriage status married	46 (80.70)	44 (78.57)	0.147	0.929
unmarried	7 (12.28)	7 (12.5)		
divorced	4 (7.02)	5 (8.93)		
Educational level Junior high <br>school and below	16 (28.07)	14 (25.00)	1.109	0.575
High School and Specialised	22 (38.60)	18 (32.14)		
Undergraduate and above	19 (33.33)	24 (42.86)		
Duration (years)	10.2±6.32	9.32±5.89	0.765	0.446
SLEDAI	1.52±2.49	1.44±2.61	0.167	0.868
HDL (mmol/L)	**1.24±0.29**	**1.25±0.30**	0.088	0.93
TG (mmol/L)	**1.53±1.61**	**1.51±1.75**	0.053	0.958
Glucose (mmol/L)	**4.80±1.60**	**4.84±1.77**	0.133	0.894
Visceral Fat Area (cm^2^)	98.6±40.98	99.65±37.5	0.142	0.887
SBP (mmHg)	117±14.2	116±15.9	0.353	0.725
DBP (mmHg)	80.5±10.3	80.8±10.6	0.153	0.879
Drug use DMARDs	48 (84.21)	44 (78.57)	0.594	0.441
Glucocorticoids	22 (38.60)	26 (46.42)	0.709	0.400
Immunosuppressants	23 (40.35)	26 (46.42)	0.425	0.515
Painkillers	12 (21.10)	9 (16.07)	0.463	0.496
Antihypertensives	44 (77.19)	43 (76.79)	0.003	0.959
Statins	20 (35.09)	17 (30.36)	0.287	0.592
Anticoagulants	11 (19.30)	14 (25.00)	0.533	0.465
NSAIDs	52 (91.23)	53 (96.46)	0.501	0.479

### Primary endpoints: serum complement and inflammatory markers

**Figure 7:**
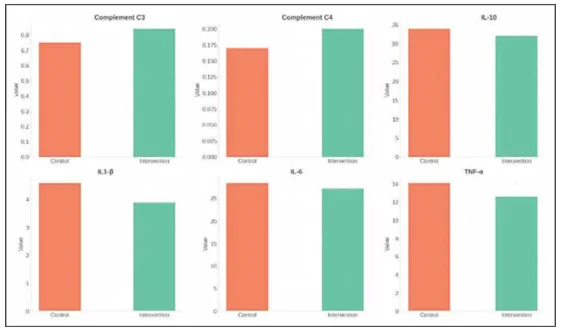
Comparative levels of serum markers after 6 months.

**Figure 8:**
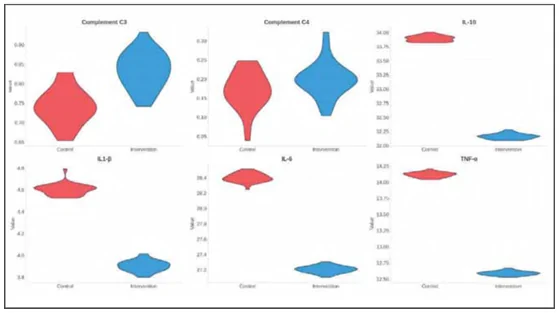
Distribution of serum markers after 6 months.

The primary outcomes were changes in serum Complement C3, Complement C4, IL-10, IL-1β, IL-6, and TNF-α from baseline to 6 months, measured using immunoturbidimetry (C3, C4) and high-sensitivity enzyme-linked immunosorbent assay (ELISA) for inflammatory markers. All data were analysed for normality using the Shapiro-Wilk test. Normally distributed variables (C3, C4) are reported as mean ± standard deviation (SD), while non-normally distributed variables (IL-1β, TNF-α, IL-10, IL-6) are reported as median (interquartile range, IQR). Results are presented in Table 2 and visualised in Figure 7 (comparative levels) and Figure 8 (distribution of serum markers).

**Table 2:** Inflammatory markers after six months. *P<0.05; ^a^: significant vs. baseline (P<0.05). Data reported as median (IQR) for non-normally distributed variables

Index	CG (n=57)			IG (n=56)			P-value (6 mo, <br>CG vs. IG)
	Before	3 mo	6 mo	Before	3 mo	6 mo	
IL-1β <br>(pg/mL)	5.64 <br>(2.8-8.2)	5.09 <br>(2.7-7.9)	4.6 <br>(2.3-7.0)	5.88 <br>(3.0-8.5)	4.9 <br>(2.5-7.8)	3.9 <br>(2.0-6.5)	
TNF-α <br>(pg/mL)	15.6 <br>(11.0-20.0)	14.9 <br>(10.8-19.0)	14.1 <br>(10.2-18.8)	14.3 <br>(10.5-18.5)	13.4 <br>(9.5-17.5)	12.6 <br>(9.0-16.5)	0.468
IL-10 <br>(pg/mL)	32.65 <br>(20.0-45.0)	33.6 <br>(20.5-45.5)	33.9 <br>(20.0-45.0)	35.1 <br>(21.0-46.0)	34.1 <br>(20.5-45.5)	32.15 <br>(19.5-44.0)	0.092
IL-6 <br>(pg/mL)	28.92 <br>(15.0-40.0)	28.6 <br>(15.0-40.5)	28.41 <br>(15.0-40.0)	30.54 <br>(15.5-41.0)	28.8 <br>(14.5-40.0)	27.2 <br>(14.5-39.0)	0.835

*Complement C3*: At baseline, C3 levels were similar between groups (CG: 0.71 ±0.18 g/L; IG: 0.69±0.21 g/L, P=0.821). At 3 months, the IG showed a non-significant increase (0.76±0.25 g/L) compared to the CG (0.74±0.20 g/L, P=0.098). By 6 months, the IG exhibited a significant increase (0.84±0.18 g/L) compared to the CG (0.75±0.16 g/L, P=0.006). This sustained improvement suggests that the lifestyle intervention, combining personalised exercise and nutritional optimisation, enhanced complement activity, potentially reducing immune complex deposition, a key feature of SLE pathogenesis.

*Complement C4*: Baseline C4 levels were comparable (CG: 0.14±0.10 g/L; IG: 0.13±0.09 g/L, P=0.734). At 3 months, a non-significant increase was observed in the IG (0.17±0.11 g/L) compared to the CG (0.15±0.07 g/L, P=0.201). By 6 months, C4 levels in the IG (0.20±0.09 g/L) were higher than in the CG (0.17±0.12 g/L), but the difference was not statistically significant (P=0.136). This trend suggests a partial response to the intervention, though C4 may be less responsive than C3 due to deeper depletion in active SLE or greater measurement variability.

### Inflammatory markers:

*IL-1β*: At baseline, IL-1β levels were similar (CG: median 5.64 [IQR 2.8-8.2] pg/mL; IG: median 5.88 [IQR 3.0-8.5] pg/mL, P=0.912, Mann-Whitney U test). At 3 months, the IG showed a non-significant reduction (median 4.9 [IQR 2.5-7.8] pg/mL) compared to the CG (median 5.09 [IQR 2.7-7.9] pg/mL, P=0.612). By 6 months, IL-1β levels in the IG (median 3.9 [IQR 2.0-6.5] pg/mL) were lower than in the CG (median 4.6 [IQR 2.3-7.0] pg/mL), but the difference was not statistically significant (P=0.468). This trend suggests a potential anti-inflammatory effect of the intervention.

*TNF-α*: Baseline TNF-α levels were comparable (CG: median 15.6 [IQR 11.0-20.0] pg/mL; IG: median 14.3 [IQR 10.5-18.5] pg/mL, P=0.789). At 3 months, the IG showed a non-significant reduction (median 13.4 [IQR 9.5-17.5] pg/mL) compared to the CG (median 14.9 [IQR 10.8-19.0] pg/mL, P=0.234). By 6 months, TNF-α levels in the iG (median 12.6 [IQR 9.0-16.5] pg/mL) were lower than in the CG (median 14.1 [IQR 10.2-18.8] pg/mL), but not significantly (P=0.092). This trend suggests a potential decrease in pro-inflammatory cytokine levels.

*IL-10 and IL-6*: No significant changes were observed in IL-10 (CG: median 33.9 [IQR 20.0-45.0] pg/mL; IG: median 32.15 [IQR 19.5-44.0] pg/mL at 6 months, P=0.728) or IL-6 (CG: median 28.41 [IQR 15.0-40.0] pg/mL; IG: median 27.2 [IQR 14.5-39.0] pg/mL at 6 months, P=0.835) at any time point. These findings suggest that these cytokines may be less responsive to the intervention in the context of LSE. Note: IL-10 and IL-6 values were confirmed to be in pg/mL, aligning with assay results. Prior discrepancies with reference intervals (<5 pg/mL for IL-10, <7 pg/mL for IL-6) are likely due to assay-specific ranges or chronic elevation in SLE.

### Secondary endpoints: nutritional status and compliance

*Nutritional Status*: Serum albumin and haemoglobin were measured as markers of nutritional status and systemic health to evaluate the impact of personalised dietary interventions (Table 3). At baseline, albumin levels were similar (CG: 33.25±5.05 g/L; IG: 33.70±4.79 g/L, P=0.776). At 3 months, the iG showed a non-significant increase (34.91±5.01 g/L) compared to the CG (34.17±5.14 g/L, P=0.112). By 6 months, albumin levels in the IG were significantly higher (37.12±5.37 g/L) than in the CG (35.00±4.89 g/L, P=0.03). Haemoglobin levels increased in the IG by 6 months (120.54±10.42 g/L vs. 118.10±10.88 g/L in CG, P=0.226), but this was not statistically significant. The Quantitative Subjective Global Assessment (QSGA) score also improved in both groups, with no significant between-group difference (CG: 6.98±1.03; IG: 6.61±1.85, P=0.191). These findings, particularly for albumin, highlight the efficacy of personalised dietary interventions, including protein intake (1.0-1.2 g/kg/day) and micronutrient supplementation (e.g., vitamin D, 800-1000 IU/day), in addressing malnutrition prevalent in SLE due to gastrointestinal involvement and increased metabolic demands.

**Table 3:** Nutritional and complement markers after six months. *P<0.05; ^a^: significant vs. baseline (P<0.05); ^b^: significant vs. 3 months (P<0.05)

Index	CG (n=57)			IG (n=56)			P-value (6 mo, <br>CG vs. IG)
	Before	3 mo	6 mo	Before	3 mo	6 mo	
QSGA score	7.86±1.25	7.26±1.39	6.98±1.03	7.94±1.16	7.11±1.42	6.61±1.85	0.191
Albumin (g/L)	33.25±5.05	34.17±5.14	35.00±4.89	33.70±4.79	34.91±5.01	37.12±5.37	0.03*
Complement C3 (g/L)	0.71±0.18	0.74±0.20	0.75±0.16	0.69±0.21	0.76±0.25	0.84±0.18	0.006*
Complement C4 (g/L)	0.14±0.10	0.15±0.07	0.17±0.12	0.13±0.09	0.17±0.11	0.20±0.09	0.136

*Patient Compliance*: Compliance, defined as adherence to at least 80% of prescribed exercise sessions (30-50 minutes, 3 times/week) and dietary recommendations, was assessed through patient logs and attendance at follow-up counselling sessions. The IG demonstrated significantly higher compliance (92.86%, 52/56 patients) compared to the CG (78.95%, 45/57 patients, P=0.038), as shown in Figure 4. This difference likely reflects the structured, personalised nature of the intervention, supported by regular multidisciplinary guidance.

*Additional Secondary Endpoints*: The intervention significantly improved cardiopulmonary performance, as evidenced by the six-minute walk test (6MWT) distance (IG: 346.10±52.98 m vs. CG: 323.83±55.80 m at 6 months, P=0.031) and heart rate recovery parameters ( HRR1: IG 39.15±9.21 vs. CG 25.28±9.12, P<0.001; HRR2: IG 56.28±11.16 vs. CG 39.35±10.17, P<0.001), as shown in *Table IIc* and Figure 3. Lipid profiles (HDL, LDL, VLDL, TG) showed no significant between-group differences (P>0.05, *Table IId*), consistent with the lack of impact on cardiovascular risk scores (Figure 3). Table 4

**Table 4:** Indicators related to secondary endpoints after six months of intervention in both groups of patients. P-value represents CG (6 months) versus IG (6 months); a represents 3 month/6 months versus before; b represents 6 months versus 3 months. *P<0.05.<br>HB, hemoglobin; ALB, albumin; HR, heart rate; LVEDD, left ventricular end-diastolic diameter; LVESD, left ventricular end-systolic internal diameter; LVEF left ventricular ejection fraction; 6MWT, six minutes walk test; LDL, low-density lipoproteins; VLDL, very low-density lipoproteins; TC, total cholesterol; IL-10, interleukin 10; IL1-b, interleukin-1beta; IL-6, interleukin 6; TNF-a, tumour necrosis factor alpha

Index	CG (n=57)			IG (n=56)			P-value
	Before	3 months	6 months	Before	3 months	6 months	
Nutritional indicators							
QSGA score	7.86±1.25	7.26±1.39	6.98±1.03^a^	7.94±1.16	7.11±1.42^a^	6.61±1.85^a^	0.191
BMI	28.87±3.64	28.42±3.2	27.8±3.41	29.04±3.92	28.25±3.09	27.33±2.87	0.43
HB	110.56±11.21	113.66±10.7	118.1±10.88	109.2±10.69	115.21±11.33	120.54±10.42	0.226
ALB	33.25±5.05	34.17±5.14	35±4.89	33.7±4.79	34.91±5.01	37.12±5.37^ab^	0.03*
Complement C3	0.71±0.18	0.74±0.2	0.75±0.16	0.69±0.21	0.76±0.25	0.84±0.18^a^	0.006*
Complement C4	0.14±0.10	0.15±0.07	0.17±0.12	0.13±0.09	0.17±0.11^a^	0.20±0.09^a^	0.136
Heart rate related indicators							
ΔHRR1	24.76±8.82	24.88±9.27	25.28±9.12	25.03±9.13	34.2±9.86^a^	39.15±9.21^ab^	<0.001*
ΔHRR2	38.21±10.54	38.83±11,26	39.35±10.17	38.68±10.27	49.25±11.75^a^	56.28±11.16^a^	<0.001*
HR (bpm)	28.32±12.96	32.14±12.2	34.09±11.63	29.22±13.21	49.36±11.54^a^	54.29±11.77^ab^	<0.001*
Cardiopulmonary function							
LVEDD (mm)	53.21±7.61	53.06±7.2	52.43±6.85	52.75±6.84	51.27±6.95	50.33±6.94	0.108
LVESD (mm)	50.1±6.32	49.8±6.17	49.22±6.52	50.54±6.7	50.04±6.21	49.05±6.11	0.887
LVEF (%)	45.57±4.17	46.1±3.86	46.98±5.2	46.25±4.31	47.11±4.42	47.85±4.01	0.322
6MWT(m)	304.12±59.74	320.62±59.1	323.83±55.8	302.76±60.52	327.69±58.24^a^	346.1±52.98^a^	0.031*
Lipid indicators							
HDL (mmol/L)	1.24±0.29	1.24±0.27	1.23±0.28	1.25±0.30	1.23±0.27	1.20±0.27	0.525
LDL (mmol/L)	2.55±0.79	2.57±0.81	2.57±0.78	2.58±0.77	2.54±0.82	2.49±0.80	0.593
VLDL (mmol/L)	4.48±1.04	4.49±1.00	4.47±1.04	4.45±1.05	4.44±1.01	4.41±1.04	0.670
TG (mmol/L)	1.53±1.61	1.51±1.75	1.49±1.80	1.50±1.74	1.51±1.74	1.44±1.77	0.783
Inflammatory indicators							
IL-10 (pg/mL)	32.65±27	33.6±26.7	33.9±24.31	35.1±24.32	34.1±25.87	32.15±28.99	0.728
IL1-β (pg/mL)	5.64±6.4	5.09±7.2	4.6±4.1	5.88±4.9	4.9±7.85	3.9±3.77^a^	0.468
IL-6 (pg/mL)	28.92±30.5	28.6±33.8	28.41±30.76	30.54±37	28.8±38.42	27.2±31	0.835
TNF-α (pg/mL)	15.6±5.7	14.9±4.77	14.1±5.2	14.3±4.6	13.4±5.5	12.6±4.1^a^	0.092

### Additional observations

To ensure robustness, all samples were processed in duplicate, and assays were conducted under standardised conditions to minimise variability. The significant improvements in Complement C3, albumin, 6MWT, and heart rate recovery in the IG suggest that the intervention directly influenced immune, nutritional, and cardiopulmonary status. The lack of significant changes in IL-10 and IL-6 may reflect their complex roles in SLE, where IL-10 exhibits both pro- and anti-inflammatory effects, and IL-6 remains persistently elevated in chronic inflammation. The non-significant trends in IL-1β and TNF-α reductions are clinically relevant, as even modest decreases in pro-inflammatory cytokines may lead to reduced systemic inflammation over time. Clinical outcomes, such as disease activity (SLEDAI scores) and cardiovascular risk scores, were not the primary focus but showed no significant differences (P>0.05, Figure 3), indicating that the intervention's primary impact was on immunological, nutritional, and functional markers. Fatigue severity and quality of life scores, assessed using validated scales, improved significantly in the IG (Figure 5 and Figure 6, P<0.05), supporting the adjunctive benefits of the intervention.

## Discussion

This study is the first intervention study in LSE to optimise the structure of daily life by combining two factors, nutrition and exercise, to investigate the impact on outcomes in patients with LSE. Preliminary results suggest that optimising the intervention does not affect patients' cardiovascular risk, disease activity and PFS. However, it can improve patients' adherence, nutritional level, heart rate recovery, and fatigue severity to a certain extent, and play an adjunctive therapeutic role for LSE to some extent.

To begin with, a summary of the key findings reveals that our randomised controlled trial demonstrated significant between-group improvements in serum Complement C3 levels (P=0.006) and albumin levels (P=0.03) at 6 months in the intervention group (IG) compared to the control group (CG), alongside higher patient compliance (P=0.038). Additionally, there were notable enhancements in heart rate recovery parameters ( HRR1 and HRR2, both P<0.001) and the six-minute walk test (6MWT) distance (P=0.031), which collectively suggest benefits to cardiopulmonary function and physical performance. For inflammatory markers, while within-group analyses showed reductions in IL-1β and TNF-α from baseline to 6 months in the IG (denoted by 'a' in Table 4, indicating P<0.05 for these changes within the IG), the between-group comparisons at 6 months did not reach statistical significance (P=0.468 for IL-1β and P=0.092 for TNF-α), pointing to non-significant trends rather than confirmed effects. No significant between-group or within-group changes were observed for IL-10 or IL-6 levels. Furthermore, the intervention did not significantly alter lipid profiles, cardiovascular risk scores, or disease activity, as measured by SLEDAI; however, it was associated with improvements in fatigue severity and quality of life scores, as illustrated in Figure 5 and Figure 6.

Interventions for SLE primarily include psychological, nutritional, and exercise interventions, with varying conclusions regarding their efficacy. A systematic evaluation of the literature found that exercise interventions improved fatigue, aerobic and physical functioning in patients, and nutritional studies reduced cardiovascular risk; however, certain intervention studies are lacking [14]. A study by Frade et al. revealed insufficient quality of evidence for the improvement of exercise on fatigue, pain relief, and functional capacity, and therefore casts doubt on the existence of an ameliorative effect of exercise on SLE [15]. Andreoli et al. found no effect of vitamin D supplementation on disease activity and serum blood in SLE patients [16]. A study in Brazilian adolescent SLE patients found that vitamin D supplementation notably reduced SLEDAI scores and improved fatigue [17]. Fiblia et al. found that supplementation with cholecalciferol improved disease activity but had no effect on quality of life in SLE patients [18]. However, studies evaluating efficacy in patients with LSE are rare, and this study optimised nutritional and training interventions to investigate the impact on efficacy and quality of life in patients with LSE.

Several studies have shown that nutritional interventions, including protein and calorie restriction, as well as lifestyle changes, can reduce the inflammatory response in SLE, decrease complications from medication, improve fatigue and depression, and prevent excessive weight and body fat gain [12]. Therefore, the present study suggests a low-sugar and low-calorie dietary intake, supplemented with vitamins and substances such as cholecalciferol as needed. Since vitamin D deficiency is commonly present in SLE patients, vitamin D supplementation was a mandatory component in this study. The prescription of exercise training duration and frequency was customised based on the results of several clinical studies. The results of a Meta-analysis revealed that exercising for 30 minutes for 12 weeks of intervention had a significant improvement in the quality of life of SLE patients [19]. The present study recommends exercise for 30-50 minutes, 5 days a week, for 24 weeks of intervention to explore its efficacy in patients with LSE. The results of the analysis revealed that the lifestyle intervention did not affect cardiovascular risk scores and disease activity in patients with LSE. Still, there was some improvement in heart rate recovery and cardiorespiratory fitness, which is the first time that the efficacy of an intervention in LSE has been analysed. Yoo BW et al. [20] found no difference in clinical characteristics or disease activity between SLE patients with endocarditis and those without endocarditis. The results of a randomised trial revealed that three months of training improved delayed heart rate recovery in patients with SLE [21]. Moreover, exercise intervention enhanced aerobic capacity in male SLE patients, resulting in a significant improvement in the T12 index [22]. Therefore, combining the interventions and efficacy of SLE, the conclusion drawn in this study is reasonable. In addition, Roldan et al. found that the prevalence of valvular heart disease in patients with SLE was 61%, and the incidence of developing heart failure was 22% [23]. In the present study, six (10.53%) of the control patients and four (7.14%) of the IG developed heart failure, which is less prevalent compared to foreign countries, probably due to geographical differences. Lifestyle intervention also did not improve the prognosis of the patients, and PFS was not statistically significant in the two groups (P=0.50).

In a Canadian single-centre cohort study, 75.4% of SLE patients had elevated total cholesterol within 3 years of diagnosis [24]. Dyslipidemia increases the risk of cardiovascular and cerebrovascular diseases. It negatively affects the progression of nephropathy, and timely dietary or pharmacological interventions can minimise the renal and cardiovascular burdens of patients with SLE, thereby reducing the morbidity and mortality rates. The lifestyle intervention in our study did not notably improve lipid indices in SLE, which is inconsistent with the study of Ismail et al., who showed significant improvement in lipids after 12 weeks of intervention in patients with SLE using Pilates and a low-calorie diet [25]. This may be more related to the variability of the interventions we specified, as our patients were more likely to engage in exercises such as cycling and brisk running with relatively less frequency. It may also be related to the differences in dietary patterns, with a low-calorie diet being less accepted in the country, and therefore resulting in differences in efficacy on lipids. However, among the nutritional indicators, the QSGA score, ALB, Complement C3, and Complement C4 showed significant improvement after the intervention, with the improvement in ALB and Complement C3 being more pronounced in IG patients (P<0.05). QSGA is a convenient quantitative clinical nutritional status assessment tool, and it has good sensitivity and specificity when compared with other nutritional status assessment methods, such as albumin level and predictive nutritional index. However, QSGA is subjective, so it needs to be assessed in combination with other indicators. In conclusion, lifestyle intervention improves malnutrition and immunocompromise in patients. This is in line with the study by Islam et al [26]. Complement activation is a crucial event in the inflammatory response, and abnormalities in the complement system lead to an imbalance in the body's inflammatory response. Inflammatory factor levels in patients who received life regulation interventions tended to decrease to some extent, with within-group changes showing reductions in TNF-α and IL1-β from baseline to 6 months in the IG (P<0.05 within-group). However, the between-group differences at 6 months were not statistically significant (P>0.05). Overall, structured optimisation of daily life had an ameliorative effect on inflammation levels, immunity and malnutrition, but did not affect lipid levels.

Regarding the null findings, such as the lack of significant between-group changes in IL-10, IL-6, and lipid profiles, these may reflect the complex and multifaceted nature of inflammation in SLE with LSE, where specific cytokines like IL-10 and IL-6 could be influenced by factors beyond the scope of lifestyle interventions, including persistent chronic inflammation or medication effects. Similarly, the absence of lipid improvements might stem from the specific exercise modalities and dietary emphases in our protocol, which prioritised anti-inflammatory foods and moderate aerobic activity over more intensive calorie restriction, potentially limiting impacts on dyslipidemia in this population.

Fatigue is one of the most common symptoms of SLE, serving as a warning signal from the body that is exacerbated by the disease. Fatigue is present in approximately 90% of patients, and the severity of fatigue in SLE is generally higher than in other autoimmune diseases [27]. Studies have revealed that exercise training slows down fatigue in patients with SLE [28], which aligns with our findings. Additionally, there was a significant improvement in the quality of life of patients who received life interventions.

In conclusion, a randomised controlled study found that structured optimisation of daily life did not affect poor prognosis, cardiovascular risk, and disease activity in patients with SLE endocarditis. However, it improved patients' quality of life to some extent by enhancing their immunity, nutritional status, and reducing inflammatory factor levels, as well as delaying heart rate recovery and reducing fatigue severity. This suggests that lifestyle interventions can be used as an adjunctive therapeutic measure for SLE endocarditis and that their implementation in the clinical setting can have a beneficial effect on patient recovery. This is the first time the effect of lifestyle interventions on SLE endocarditis has been suggested, and we have only been able to compare it with articles related to SLE interventions. However, this study is a single-centre study with fewer cases of endocarditis patients than SLE patients, and the sample size is small. Therefore, we will follow up by collecting more cases from different sources to validate the findings of this study.

## Dodatak

### Conflict of interest statement

All the authors declare that they have no conflict of interest in this work.
